# INTERGROWTH-21st Gestational Dating and Fetal and Newborn Growth Standards in Peri-Urban Nairobi, Kenya: Quasi-Experimental Implementation Study Protocol

**DOI:** 10.2196/10293

**Published:** 2018-06-22

**Authors:** Kathryn Millar, Suha Patel, Meghan Munson, Linda Vesel, Shalini Subbiah, Rachel M Jones, Sarah Little, Aris T Papageorghiou, Jose Villar, Mary Nell Wegner, Nick Pearson, Faith Muigai, Catherine Ongeti, Ana Langer

**Affiliations:** ^1^ Department of Family Health Care Nursing School of Nursing University of California, San Francisco San Francisco, CA United States; ^2^ Division of Global Obstetrics and Gynecology Brigham and Women's Hospital Boston, MA United States; ^3^ Jacaranda Health Nairobi Kenya; ^4^ Women and Health Initiative Department of Global Health and Population Harvard T.H. Chan School of Public Health Boston, MA United States; ^5^ Oxford Maternal & Perinatal Heath Institute Nuffield Department of Obstetrics & Gynaecology University of Oxford Oxford United Kingdom; ^6^ PharmAccess Foundation-SafeCare Nairobi Kenya

**Keywords:** ultrasound, gestational age, fetal growth, health care quality, anthropometry

## Abstract

**Background:**

The burden of preterm birth, fetal growth impairment, and associated neonatal deaths disproportionately falls on low- and middle-income countries where modern obstetric tools are not available to date pregnancies and monitor fetal growth accurately. The INTERGROWTH-21^st^ gestational dating, fetal growth monitoring, and newborn size at birth standards make this possible.

**Objective:**

To scale up the INTERGROWTH-21^st^ standards, it is essential to assess the feasibility and acceptability of their implementation and their effect on clinical decision-making in a low-resource clinical setting.

**Methods:**

This study protocol describes a pre-post, quasi-experimental implementation study of the standards at Jacaranda Health, a maternity hospital in peri-urban Nairobi, Kenya. All women with viable fetuses receiving antenatal and delivery services, their resulting newborns, and the clinicians caring for them from March 2016 to March 2018 are included. The study comprises a 12-month preimplementation phase, a 12-month implementation phase, and a 5-month post-implementation phase to be completed in August 2018. Quantitative clinical and qualitative data collected during the preimplementation and implementation phases will be assessed. A clinician survey was administered eight months into the implementation phase, month 20 of the study. Implementation outcomes include quantitative and qualitative analyses of feasibility, acceptability, adoption, appropriateness, fidelity, and penetration of the standards. Clinical outcomes include appropriateness of referral and effect of the standards on clinical care and decision-making. Descriptive analyses will be conducted, and comparisons will be made between pre- and postimplementation outcomes. Qualitative data will be analyzed using thematic coding and compared across time. The study was approved by the Amref Ethics and Scientific Review Committee (Kenya) and the Harvard University Institutional Review Board. Study results will be shared with stakeholders through conferences, seminars, publications, and knowledge management platforms.

**Results:**

From October 2016 to February 2017, over 90% of all full-time Jacaranda clinicians (26/28) received at least one of the three aspects of the INTERGROWTH-21^st^ training: gestational dating ultrasound, fetal growth monitoring ultrasound, and neonatal anthropometry standards. Following the training, implementation and evaluation of the standards in Jacaranda Health’s clinical workflow will take place from March 2017 through March 5, 2018. Data analysis will be finalized, and results will be shared by August 2018.

**Conclusions:**

The findings of this study will have major implications on the national and global scale up of the INTERGROWTH-21^st^ standards and on the process of scaling up global standards in general, particularly in limited-resource settings.

**Registered Report Identifier:**

RR1-10.2196/10293

## Introduction

### Background

The neonatal period (first 28 days of life) is the most vulnerable time for an infant’s survival. In 2016, 2.6 million neonates died globally representing 46% of all under-five deaths [1]. Most of these deaths took place in low- and middle-income countries (LMICs), including 80% in sub-Saharan Africa and South Asia [1]. Preterm birth related complications are the primary cause of neonatal deaths [1]. A common condition associated with preterm birth is low birth weight (LBW; <2.5 kg), which contributes to 60-80% of neonatal deaths [2] and can lead to long-term complications, including developmental delays [2-7]. There are two causes of LBW in neonates: prematurity, growth restriction during pregnancy resulting in a birthweight that is small for gestational age (SGA) [8], or a combination of the two conditions. SGA newborns have nearly twice the risk of neonatal and post-neonatal mortality [3] and account for 21.9% of neonatal deaths in LMICs [9].

To make sound clinical decisions and provide quality maternal and neonatal care, clinicians require measurement standards for accurate pregnancy dating, monitoring of fetal growth, and assessment of newborn size and growth [8]. Such standards enable clinicians to provide appropriately-timed antenatal care (ANC) [10]; identify the need for high-risk ANC consultations and referrals; and anticipate, identify, and manage fetal growth restriction [10,11], preterm labor [12,13], and maternal and neonatal [12] complications effectively [14]. The ability to date a pregnancy accurately affects a clinician’s ability to make informed decisions about appropriate timing of labor induction for maternal and fetal indications (including prolonged gestation) and elective or repeat caesarean sections [15]. Accurate gestational dating is also essential for birth preparedness, allowing women to plan for transportation to a birth facility at the appropriate time [16]. Lastly, at the time of birth, correctly assessing the neonate’s size for gestational age is critical for appropriate clinical management and potential referral for abnormal size and growth [17].

The date of the maternal last menstrual period (LMP) can be used for pregnancy dating but is often inaccurate or unknown; in these cases, ultrasound is the most accurate method for determining gestational age [12,18-20]. Ultrasound is also the gold standard for monitoring fetal growth disturbances [21]. However, ultrasound equipment is not always available and fully functional in low-resource settings; even when it is, there is often a lack of trained personnel with the technical knowledge to use it effectively [22,23]. As a result, gestational age is often not determined, which makes monitoring and intervention for poor fetal growth impossible [22,23]. Because of this, the standard for assessing gestational age and fetal growth in most LMICs is date of LMP [12] and fundal height [24], respectively. The literature has shown that both methods have a high margin of error, which can lead to inaccurate dating, diagnoses, and clinical management [25-27].

In 2014, the INTERGROWTH-21^st^ Project completed a five-year, global, prospective study of growth, health, nutrition, and neurodevelopment. The study followed women and their infants longitudinally from less than 14 weeks gestation until two years postnatal. The project enrolled populations at low risk of adverse outcome in Brazil, Italy, Oman, UK, USA, China, India, and Kenya [28,29]. INTERGROWTH-21^st^ data were compiled to develop new prospective standards to be used to assess pregnancy dating in the first trimester [30] and second trimester [19], fetal growth [11] (including fundal height [24] and ultrasound assessment [11]), and newborn size at birth [13,17]. With these evidence- and globally-based standards, for the first time, clinicians will be able to monitor growth based on how healthy babies in any population should grow [14]. These standards complement the World Health Organization (WHO) Child Growth Standards, together offering a standardized method to assess growth throughout the continuum of fetal life through early childhood development, which is useful for both clinicians and patients.

To scale these standards and affect preterm birth and its complications, including neonatal mortality, it is essential to assess the feasibility and acceptability of implementing these standards and their effect on clinical decision-making, particularly in low-resource clinical settings.

### Aim

We aim to assess the feasibility and acceptability of implementing the INTERGROWTH-21^st^ standards over a one-year period at Jacaranda Health, a private, social enterprise maternity hospital in peri-urban Nairobi, Kenya. To our knowledge, this is the first facility-based implementation study of the INTERGROWTH-21^st^ standards in a limited-resource setting. The research design considers stakeholder inputs, the physical and institutional environment, and the health system structure.

The ultimate aim of the project is to use the results of the study to inform the implementation and scale up of the INTERGROWTH-21^st^ standards in other settings and to inform the translation of guidelines and tools into routine clinical practice.

### Primary Objective

The primary objective is to determine the facilitators and barriers to implementing the INTERGROWTH-21^st^ standards at Jacaranda Health. The specific components of the primary objective are to assess the introduction of the INTERGROWTH-21^st^ standards and the training of clinicians at Jacaranda Health, the effect of the implementation of the INTERGROWTH-21^st^ standards on clinical practices at Jacaranda Health, clinicians’ experiences and satisfaction with the INTERGROWTH-21^st^ standards, clinicians’ perceived effect of the standards on the clinical care they provide, and patient experiences and satisfaction with the care they received at Jacaranda Health during implementation of the INTERGROWTH-21^st^ standards.

### Secondary Objective

The secondary objective of this study is to assess the effect of implementing the INTERGROWTH-21^st^ standards on clinicians’ decision-making and patient outcomes, including the processes for determining gestational age and estimated due date, internal referral to Jacaranda Health clinicians providing high-risk consultations, tertiary-center referral of high-risk pregnant women, and indications for and rates of labor inductions and caesarean sections.

## Methods

### Study Design

This is a pre-post, quasi-experimental implementation study using quantitative clinical data, focus group discussions (FGDs), in-depth interviews (IDIs), and a short clinician survey. The study describes the feasibility, acceptability, and the effect of implementing the INTERGROWTH-21^st^ standards on clinical decision-making and management at Jacaranda Health. Study activities consisted of a 12-month preimplementation phase which included a baseline facility assessment, changes to facility protocols and charting forms, planning work flow adaptations to facilitate implementation of the standards, equipment procurement, training of clinicians, and baseline data collection; a 12-month implementation phase which included the implementation of the INTERGROWTH-21^st^ clinical standards, revised facility protocols and charting forms into routine clinical practice, and data collection; and a five-month post-implementation phase which includes analysis and dissemination.

This study design was based on a conceptual model created by INTERGROWTH-21^st^ researchers at Oxford University and researchers at the Harvard T.H. Chan School of Public Health.

### Study Setting

This implementation study is currently being carried out at Jacaranda Health, a social enterprise, 18-bed maternity hospital that provides women in peri-urban Nairobi with affordable, safe, and respectful ANC, standard vaginal and cesarean delivery, and postnatal care (PNC) services. Women in preterm labor and newborns with LBW and/or complications are not managed at this facility and are referred to tertiary-level facilities for specialized care. With a model that emphasizes quality and affordable care provided primarily by nurse-midwives (and supported by a team of highly skilled physicians, clinical educators, and managers), Jacaranda Health provides an ideal venue for evaluating the implementation of the INTERGROWTH-21^st^ standards and capturing factors that facilitate and challenge that process. Jacaranda Health patients come from densely-populated, peri-urban neighborhoods in northeastern Nairobi, including Kiambu, Thika, Gatundu, and Embakasi districts. These areas are served by many facilities that range from small pharmacies and outpatient care clinics to private and public sector secondary and tertiary hospitals with maternity wards; the services and prices vary substantially across these facilities.

### Study Population

Pregnant women with a viable fetus presenting for ANC and/or delivery at Jacaranda Health were eligible for the following three elements of the intervention: (1) gestational dating standards for women who present for their initial ANC visit in the first or second trimester (more than eight and less than or equal to 26 weeks gestation); (2) fetal growth monitoring standards for women who present for an ANC visit in the third trimester (after 26 weeks gestation) and are identified as high-risk based on factors related to their surgical, medical, or obstetric history or current pregnancy; and (3) newborn size at birth standards for all newborns born at Jacaranda Health. We excluded pregnant women with a nonviable fetus in both quantitative and qualitative data collection, women who present for their initial ANC visit in the third trimester (after 26 weeks gestation) for gestational dating standards, and parents of stillborn infants in qualitative data collection. All eligible pregnant women and mothers of newborns described above were eligible to participate in FGDs and IDIs.

All clinicians delivering ANC and newborn anthropometry who attended INTERGROWTH-21^st^ training sessions are eligible to participate in FGDs, IDIs, and a short clinician survey. Clinicians who only work in child wellness clinics and not in prenatal or intrapartum care units were excluded.

### Patient and Public Involvement

The development of the study design, research questions, and outcome measures did not formally involve patient and public opinions and contributions. However, the study assesses patient and provider perceptions and experiences of the implementation of the INTERGROWTH-21^st^ standards at Jacaranda Health to assess their acceptability and inform their further scale up. The results of the study will be disseminated to providers at Jacaranda Health by study staff at the end of the study.

### Preimplementation Phase

#### Baseline Facility Assessment

Before implementing the INTERGROWTH-21^st^ standards, we conducted a baseline assessment of current facility practices as they relate to our study objectives. Focus was placed on policies and practices related to ANC provision, pregnancy dating, identification and referral of high-risk pregnancies to high-risk care within Jacaranda Health and to tertiary facilities, fetal growth monitoring, newborn size measurement, and indications for and rates of cesarean section and labor induction.

The baseline assessment was conducted through clinic observations; a desk review of written policies; IDIs with Jacaranda Health’s director of clinical operations, clinical programs manager, and clinical educator; a chart review to understand clinician practices and indications for and rates of cesarean sections and labor inductions; an equipment and supply inventory with a focus on ultrasound and newborn anthropometry; and a human resource inventory to understand existing personnel and clinician roles and responsibilities related to clinic flow, pregnancy dating, fetal growth monitoring, and patient counseling. We then adapted ANC protocols to align with the 2002 WHO ANC model [31]. This work was done prior to the release of the 2016 WHO ANC recommendations [32].

#### Protocol, Charting, Equipment, and Work Flow Adaptations

A key element of preimplementation activities was ensuring that the hospital’s equipment, protocols, and procedures were updated and adapted for the implementation of the INTERGROWTH-21^st^ standards. In partnership with Oxford University, we created a computerized calculator to calculate gestational age and fetal growth percentiles: clinicians use the ultrasound machine to measure the required biometrics for gestational dating or fetal growth, input the measurements into the calculator, and then record the resulting gestational age or fetal growth percentiles in the patient’s chart. Additionally, neonatal scales, measuring tapes, and infantometers, chosen in consultation with the INTERGROWTH-21^st^ Oxford team and adjusted based on local availability and resource constraints, were sourced and integrated into clinical practice.

We updated Jacaranda Health clinical protocols for standard ANC, high-risk pregnancy classification and subsequent internal and tertiary-center referral, gestational dating, fetal growth monitoring, and newborn anthropometry, in addition to corresponding patient charting forms, to support the implementation and evaluation of the INTERGROWTH-21^st^ standards.

Clinician job aids were created to facilitate the implementation of the standards and related decision-making algorithms. We also altered clinic flow processes to accommodate the introduction of ultrasound services; a separate room was designated exclusively for gestational dating ultrasounds. In the context of adapting clinical definitions of high-risk pregnancy criteria and processes for both internal and tertiary-center referrals, we trained clinicians to only do activities (like ultrasound) within their scope of practice as determined by the Nursing Council of Kenya.

#### Training

A main aim of implementation was training Jacaranda Health staff how to use the INTERGROWTH-21^st^ standards. This was done in collaboration with the original INTERGROWTH-21^st^ Project study team based at Oxford University and Aga Khan University Hospital (AKUH) in Nairobi, Kenya [33-37].

From October 2016 to February 2017, we conducted training of Jacaranda Health clinical and management staff on the purpose and use of the INTERGROWTH-21^st^ gestational dating ultrasound, fetal growth monitoring ultrasound, and neonatal anthropometry standards. Emphasis was placed on including assessment, identification, and referral of high-risk patients as part of the study design to evaluate operational system capacity to support the implementation of the standards and their clinical implications.

An obstetrician-led half-day training included essential components of ANC, basic obstetric ultrasound skills, and the INTERGROWTH-21^st^ standards and accompanying adaptations to charting practices and clinical protocols. Of the 23 participants in the training, 22 were nurse-midwives and one was a clinical officer; these staff provide the majority of ANC and PNC at Jacaranda Health.

An obstetrician and an INTERGROWTH-21^st^ anthropometry trainer from AKUH provided a half-day anthropometry training which included theory, equipment, and techniques needed to perform accurate newborn length, weight, and head circumference measurements. The anthropometry trainer certified clinicians in neonatal anthropometry after they performed length, weight, and head circumference measurements accurately on newborns. One group of 24 clinicians, 23 nurse-midwives and one clinical officer, attended this training. Nearly 90% (24/28) of all full-time and part-time Jacaranda Health clinicians attended these initial trainings.

AKUH trained Jacaranda Health’s primary sonographer in INTERGROWTH-21^st^ ultrasound measurements for the first, second, and third trimesters over the course of two weeks. At the end of the two weeks, the sonographer demonstrated proficiency in performing INTERGROWTH-21^st^ measurements as determined by a senior radiologist at AKUH. The Jacaranda Health sonographer then trained a group of six Jacaranda Health nurse-midwives with prior experience in basic obstetric ultrasound during a half-day training on gestational dating ultrasounds. Three of the six nurse-midwives were certified to perform gestational dating ultrasounds after proving competence in performing measurements and calculating estimated delivery date accurately three consecutive times in the presence of the sonographer. This group of three nurse-midwives constitutes over 10% (3/28) of all clinicians.

### Implementation Phase

After training was completed and equipment was put into place, the INTERGROWTH-21^st^ standards, revised facility protocols, and adapted patient charting forms were introduced into routine clinical practice in March 2017. The clinic work flow adaptations were implemented to facilitate the identification of pregnant women eligible for ultrasound using the new standards and ensure that pregnant women were seen by the appropriate clinician. Work flow adaptations were not needed to implement the newborn size at birth standards since newborn anthropometry was an established part of routine practice.

#### Quality Monitoring

To ensure the quality of the implementation of the INTERGROWTH-21^st^ standards, three quality monitoring processes were utilized: expert ultrasound image review, weekly clinic stakeholder meetings, and targeted refresher training. Our protocol includes sending copies of de-identified gestational dating and fetal growth ultrasound images to the quality assurance team at Oxford University. The team reviews the images, assesses the quality of each image based on INTERGROWTH-21^st^ guidelines, and provides guidance to Jacaranda Health staff on how to improve ultrasound quality, if needed. Images were sent to Oxford for review every two months via a double password-protected Dropbox folder. The program management team at Jacaranda Health then shared feedback from the Oxford team with the ultrasound providers to strengthen sonography skills and processes. Reinforcement training was provided by Oxford University clinical researchers specializing in sonography midway through implementation to further improve the quality of the ultrasound procedures. Challenges in clinical implementation were discussed by the clinical staff during weekly meetings, which enabled staff to quickly resolve any problems. Lastly, through chart review and observation, the clinical and project management teams had the discretion to identify clinicians who required targeted refresher training and to provide that training at any point during the study. Important project notifications and reminders were administered to all staff at weekly clinical meetings by clinic managers and project management.

### Study outcomes

#### Primary outcomes

The primary study outcomes are (1) clinicians’ and patients’ perception of facilitators and barriers to the implementation of the INTERGROWTH-21^st^ standards and (2) uptake of gestational dating ultrasounds, fetal growth monitoring by ultrasound, and newborn anthropometry. These outcomes were explored through the following dimensions: feasibility, acceptability, appropriateness, adoption, fidelity and penetration [38].

#### Secondary outcomes

The secondary outcomes used to evaluate clinical decision-making include (1) proportion of ANC clients whose gestational age and estimated due date were correctly calculated and documented, (2) proportion of high-risk pregnant women who were referred internally to a high-risk clinician or to a tertiary care facility, (3) proportion of pregnant women receiving gestational dating scans who were induced for labor due to a prolonged pregnancy, and (4) proportion of pregnant women receiving gestational dating scans who delivered via cesarean section.

#### Data collection

During the preimplementation phase, baseline data were collected for 12 consecutive months (months 1-12) prior to the start of the implementation phase. Data were also collected for 12 consecutive months during the implementation phase (months 13-24). These preimplementation and implementation phase quantitative data come from patient charting forms completed in the two-year period. Qualitative data were also collected during the preimplementation phase (month eight) and the implementation phase (month 16 and month 24). Additionally, a one-time short provider survey was administered during the implementation phase at month 20 to assess clinicians’ attitudes and acceptability of the standards. (Table 1).

#### Quantitative Data Collection

We collected outcome data from patient charts and from a clinic log of external referrals for pregnancies and deliveries one year before and one year after the start of implementation.

**Table 1:** Data collection tools and timeline.

Method and tool		Preimplementation (months 1-12)		Implementation (months 13-24)			
		Month 8	Months 1-12	Month 16	Month 20	Month 24	Months 13-24
**Quantitative**							
	Chart review		X				X
	Clinic referral log		X				X
**Qualitative**							
	Patient in-depth interviews	X		X		X	
	Patient focus group discussions	X		X		X	
	Clinician in-depth interviews	X		X		X	
	Clinician focus group discussions	X		X		X	
	Clinician survey				X		

#### Qualitative Data Collection

We conducted FGDs and IDIs with participants sampled from two population groups using purposeful and convenience sampling: (1) patients who received ANC and/or delivery care at Jacaranda Health and (2) clinicians who work directly with patients (providing ANC, delivery care, or anthropometry at birth) including nurse-midwives, hospital managers, ultrasonographers, and physicians.

FGDs and IDIs with patients and clinicians were conducted in the preimplementation phase and twice during the implementation phase. Patients were interviewed to evaluate their perceptions, attitudes, and experiences of receiving an ultrasound for gestational dating and fetal growth, their newborn receiving a growth assessment, and their interactions with clinicians implementing this care. Clinicians were interviewed to evaluate their perceptions, attitudes, and experiences of the introduction and implementation of the new standards as part of their routine clinical practice.

The research team developed semistructured discussion guides for both FGDs and IDIs, which were piloted with Jacaranda Health staff and patients. Research assistants conducted all interviews in a private and secure location and took great care to protect the identity and confidentiality of all participants. To encourage patient participation in qualitative interviews, we offered reimbursement for transportation to the facility and free refreshments after the completion of the interviews. All FGDs and IDIs were audio-recorded and transcribed verbatim by an external transcriber; in the instances that participants spoke a language other than English, the transcriber translated the recording to English for the transcript. Hand-written notes taken by the research assistant provided the context for the interviews.

Additionally, a survey (10 questions) was administered by a research assistant to all clinicians during the implementation phase at month 20 via Survey Monkey on an Android tablet. Clinicians were asked to grade their comfort (using a Likert scale) with the INTERGROWTH-21^st^ standards, the ease of integration into their workflow, and their perceptions of the effect of the standards on the quality of care they provided.

#### Sample Size

We used a census of clinicians and patients for this implementation study based on the inclusion and exclusion criteria described prior. According to clinic estimates and projections of ANC and delivery care utilization, data for up to 5,000 pregnant women and newborns will be recorded. The total number included in the final analysis will be based on the number of patients who meet eligibility criteria as confirmed at the time of data entry based on indicators in patient charting forms.

In each time period, the sample will be stratified into the following three categories: (1) pregnant women who attended ANC at Jacaranda Health and delivered there, allowing analysis of longitudinal ANC and delivery data; (2) pregnant women who attended ANC at Jacaranda Health but did not deliver there, providing ANC data only; and (3) women who did not attend ANC but who delivered at Jacaranda Health, capturing delivery and newborn data only. All clinic managers, physicians, and nurse-midwives who met the criteria are included. Clinician attrition will be documented.

### Postimplementation Phase

#### Data Analysis and Management

##### Quantitative Data

Data from patient charts and referral logs were double-entered and managed in REDCap [39], a secure online data capture system with built-in data entry restrictions, data quality tools, and protection of personally identifiable information. Quality control measures have been in place to check the data at various stages on a routine basis using REDCap [39] and Stata 15 [40]. REDCap’s data quality features were utilized to ensure that data was entered within acceptable ranges and in the proper formats [39]. Additionally, data were checked for consistency and errors using a Stata 15 [40] script. All discrepancies have been resolved by checking original paper charts.

Most implementation and process indicators will be measured and presented using descriptive statistics. We will analyze changes in quantitative outcomes where relevant, by evaluating the difference in response to the indicators between pre- and postimplementation. Measurements of differences in continuous data will be assessed using t-tests for data that is normally distributed; otherwise, a non-parametric test will be performed. Categorical data will be compared using a chi-squared test. Quantitative data will be analyzed using Stata 15 [40]. All data will be de-identified before analysis begins.

##### Qualitative Data

For each cycle of qualitative data transcription, the transcriber completed transcription of one initial data file and sent it to the qualitative data manager at Jacaranda Health for quality control prior to transcribing the rest of the data files.

Qualitative data collected at each stage are being analyzed independently by two investigators using thematic coding in NVivo [41] and compared across time. Transcribed, de-identified qualitative data are being stored in a double password-protected Dropbox folder accessible only to a select number of study personnel.

#### Dissemination Policy

Results of the study will be shared with key stakeholders both in Kenya and globally through a national dissemination meeting, global conferences, an online knowledge management platform, and publications.

### Ethical Considerations

The Amref Ethics and Scientific Review Committee of Kenya and the Harvard University Institutional Review Board approved all study activities, protocols, and standards prior to the commencement of study activities.

Facility-level informed consent was obtained from the Jacaranda Health hospital manager, acting as the facility’s representative, prior to the collection of any implementation data. The facility informed consent emphasized that no patient-identifiable health data would be shared or disseminated beyond the Jacaranda Health team. All FGD, IDI, and survey participants—patients and clinicians—provided written informed consent prior to any interview. Participants were informed that they could withdraw their consent at any time and be removed from the sample.

## Results

From October 2016 to February 2017, over 90% of all Jacaranda Health clinicians (25 nurse-midwives and one clinical officer of 28 total Jacaranda Health clinicians) received at least one of the three aspects of the INTERGROWTH-21^st^ training: gestational dating ultrasound, fetal growth monitoring ultrasound, and neonatal anthropometry standards. Following the training, the implementation and study of the INTERGROWTH-21^st^ standards as part of Jacaranda’s clinical workflow took place from March 2017 through March 5, 2018. Data analysis will be finalized, and results are expected in August 2018.

## Discussion

### Significance

The findings of this study will have major implications on the national and global scale up of the INTERGROWTH-21^st^ standards and on the process of scaling up global standards in general, particularly in limited-resource settings. The ability to implement a standard methodology of gestational dating, fetal growth monitoring, and assessment of newborn size at birth will result in better data to enable clinicians, researchers, and policy makers to more accurately identify and quantify high-risk pregnancies, preterm birth, and fetal and neonatal growth disturbances. In turn, standardized data that is comparable across global populations empowers researchers and policy makers to better understand and act on distributions of high-risk pregnancies, restricted growth, and prematurity. Lastly, based on this data, clinical practice and resources can be modified to meet the needs of pregnant women and their fetuses and newborns to decrease the incidence and morbidities associated with poor fetal and newborn growth and prematurity.

### Limitations

While Jacaranda Health provides the right environment for this study, the generalizability of our findings needs to be carefully considered when applying lessons learned to other clinical settings. Given that clinic protocols were amended to meet the 2002 WHO ANC guidelines, which affected the criteria for high-risk referral, this change may confound the outcome of the percentage of women who are referred to high-risk care. The study is also limited in its ability to assess the long-term health outcomes of these standards, yet it will inform future research designed to assess them.

### Strengths

This will be the first study to assess the feasibility and acceptability of introducing the new INTERGROWTH-21^st^ clinical standards into a low-resource setting. Due to the dearth of innovative gestational dating tools and burden of preterm birth in low-resource settings, it is particularly important to study the implementation of these standards in this setting. The results of our evaluation will provide useful insights and recommendations for further implementation in similar clinical settings in Kenya and beyond.

Additionally, the mixed-methods approach used in this study will yield unique insights into the barriers, facilitators, and process of implementing the INTERGROWTH-21^st^ standards and the resulting impact on short-term clinical decision-making. Lastly, Jacaranda Health is dedicated to health facility quality improvement in peri-urban Nairobi, which makes it an excellent venue for evaluating the implementation of the INTERGROWTH-21^st^ standards and for capturing factors that facilitate and challenge that process.

## Abbreviations

AKUH: Aga Khan University Hospital
ANC: antenatal care
FGD: focus group discussion
IDI: in-depth interview
LBW: low birth weight
LMICs: low- and middle-income countries
LMP: last menstrual period
PNC: postnatal care
SGA: small for gestational age
WHO: World Health Organization
